# The interplay between redox signalling and proteostasis in neurodegeneration: *In vivo* effects of a mitochondria-targeted antioxidant in Huntington's disease mice

**DOI:** 10.1016/j.freeradbiomed.2019.11.021

**Published:** 2020-01

**Authors:** Brígida R. Pinho, Ana I. Duarte, Paula M. Canas, Paula I. Moreira, Michael P. Murphy, Jorge M.A. Oliveira

**Affiliations:** a REQUIMTE/LAQV, Department of Drug Sciences, Pharmacology Lab, Faculty of Pharmacy, University of Porto, Porto, Portugal; b CNC - Center for Neuroscience & Cell Biology, University of Coimbra, Coimbra, Portugal; c Institute for Interdisciplinary Research (IIIUC), University of Coimbra, Coimbra, Portugal; d Institute of Physiology, Faculty of Medicine, University of Coimbra, Coimbra, Portugal; e MRC Mitochondrial Biology Unit, University of Cambridge, Cambridge, CB20XY, UK; f Consortium for Mitochondrial Research (CfMR), University College London, Gower Street, WC1E 6BT, London, UK

**Keywords:** Mitochondria, Huntington's disease, Oxidative stress, Antioxidant, Neurodegeneration, Unfolded protein response, ALS, amyotrophic lateral sclerosis, BSA, bovine serum albumin, HD, Huntington's disease, Htt, huntingtin, mHtt, mutant huntingtin, MitoQ, mitoquinone or MitoQ_10_, mtDNA, mitochondrial DNA, UPR^mt^, mitochondrial unfolded protein response, ROS, reactive oxygen species, SDHA, succinate dehydrogenase complex, subunit A, SOD2, superoxide dismutase 2, TFAM, mitochondrial transcription factor A, UPS, ubiquitin-proteasome system, WT, wild-type

## Abstract

Abnormal protein homeostasis (proteostasis), dysfunctional mitochondria, and aberrant redox signalling are often associated in neurodegenerative disorders, such as Huntington's (HD), Alzheimer's and Parkinson's diseases. It remains incompletely understood, however, how changes in redox signalling affect proteostasis mechanisms, including protein degradation pathways and unfolded protein responses (UPR). Here we address this open question by investigating the interplay between redox signalling and proteostasis in a mouse model of HD, and by examining the *in vivo* effects of the mitochondria-targeted antioxidant MitoQ. We performed behavioural tests in wild-type and R6/2 HD mice, examined markers of oxidative stress, UPR activation, and the status of key protein degradation pathways in brain and peripheral tissues. We show that R6/2 mice present widespread markers of oxidative stress, with tissue-specific changes in proteostasis that were more pronounced in the brain and muscle than in the liver. R6/2 mice presented increased levels of cytosolic and mitochondrial chaperones, particularly in muscle, indicating UPR activation. Treatment with MitoQ significantly ameliorated fine motor control of R6/2 mice, and reduced markers of oxidative damage in muscle. Additionally, MitoQ attenuated overactive autophagy induction in the R6/2 muscle, which has been associated with muscle wasting. Treatment with MitoQ did not alter autophagy markers in the brain, in agreement with its low brain bioavailability, which limits the risk of impairing neuronal protein clearance mechanisms. This study supports the hypotheses that abnormal redox signalling in muscle contributes to altered proteostasis and motor impairment in HD, and that redox interventions can improve muscle performance, highlighting the importance of peripheral therapeutics in HD.

## Highlights

• R6/2 mice display behavioural impairments, oxidative stress and altered proteostasis.
• R6/2 mice show tissue-specific cytosolic & mitochondrial unfolded protein responses.
• R6/2 muscle has increased oxidative markers, ubiquitination, and autophagy activation.
• MitoQ reduces autophagy in R6/2 muscle but not brain, where bioavailability is lower.
• MitoQ ameliorates fine motor control through reduction of oxidative damage in muscle.

## Graphical abstract

**Figure:**
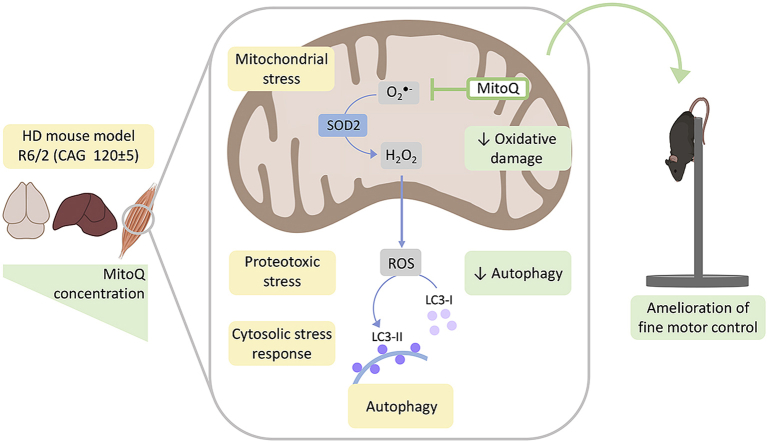

## 1 Introduction

Neurodegenerative disorders often present common pathological mechanisms, including abnormal protein homeostasis (proteostasis), mitochondrial dysfunction, and aberrant redox signalling [1,2]. Physiological redox signalling involves the reversible oxidation of specific protein residues by reactive oxygen species (ROS), such as the H_2_O_2_ generated from superoxide produced in mitochondria and by NADPH oxidases [3,4]. The interplay between redox signalling and proteostasis is a subject under active discussion. Disturbed proteostasis and the associated proteotoxic stress may trigger a loss of redox homeostasis [5]. Conversely, aberrant redox signalling may compromise the normal activity of key proteostasis effectors such as molecular chaperones [6,7] and protein degradation pathways, including the ubiquitin-proteasome system (UPS) [8] and autophagy [9]. In the context of neurodegeneration, it remains incompletely understood how changes in redox signalling modulate proteostasis, particularly cytosolic and mitochondrial unfolded protein responses (UPR), and also how targeted interventions that prevent localized ROS production in mitochondria affect proteostasis. A better understanding of this interplay should contribute to develop effective treatments.

Huntington's disease (HD) is a monogenic neurodegenerative disorder that has been increasingly recognized as a systemic disease, due to the presence of several peripheral pathologies in HD patients [10,11]. HD is caused by a CAG repeat expansion in the first exon of the huntingtin (Htt) gene, determining the formation of a mutant Htt protein (mHtt) with an elongated N-terminal polyglutamine stretch. mHtt is prone to proteolytic cleavage, misfolding and aggregation [11,12]. Key pathogenic mechanisms in HD present similarities with other neurodegenerative disorders. The accumulation and aggregation of mHtt disturbs the proteostasis network [13], mHtt induces abnormalities in mitochondrial dynamics and quality control [14], and oxidative biomarkers are increased in HD patients and animal models [[15], [16], [17]], suggesting aberrant redox signalling. The well-defined genetic cause and the pathological features similar to other neurodegenerative disorders support the selection of HD as a model-disease to study the interplay between redox signalling and proteostasis. Related to this interplay, we recently showed an increase of mHtt aggregation upon enhancement of mitochondrial superoxide generation in a HD cell model [18].

Mitochondrial superoxide is a major source of ROS, playing a key role in redox signalling [19,20]. MitoQ_10_ (MitoQ) or mitoquinone is a lipophilic, positively-charged chain-breaking antioxidant that accumulates in mitochondria [21], where it may contribute to attenuate maladaptive ROS-induced ROS release cycles [22]. MitoQ has been tested in models of neurodegenerative disorders, including spinocerebellar ataxia [23], amyotrophic lateral sclerosis (ALS) [24], Parkinson's [25] and Alzheimer's diseases [26], where it was found to reduce oxidative damage and ameliorate disease phenotypes. However, in these previous studies the effects of MitoQ on cellular proteostasis were not addressed. Moreover, while ROS are early inducers of autophagy [9] and non-targeted antioxidants, such as N-acetylcysteine and vitamin E, inhibited autophagy in cellular and small-organism HD models [27], it remains uncertain what is the impact of mitochondria-targeted antioxidants on proteostasis mechanisms, including autophagy. In this context, the mitochondria-targeted antioxidant MitoQ is an interesting model-drug to assess the effects of ROS modulation on proteostasis.

In the present study we investigate the interplay between redox signalling and proteostasis in the R6/2 HD mouse model and wild-type controls. We characterize the behavioural phenotype of the HD mice and examine the association with markers of oxidative stress, activation of unfolded protein responses, and the status of key protein degradation pathways in the brain and peripheral tissues. We find that while R6/2 mice present widespread markers of oxidative stress, changes in proteostasis are more pronounced in the brain and muscle than in the liver. We test the *in vivo* effects of MitoQ and find that it ameliorates fine motor control by reducing markers of oxidative damage, particularly in the muscle where it shows higher bioavailability. MitoQ also attenuates ROS-induced muscle autophagy, without changing autophagy markers in the brain, where MitoQ has lower bioavailability. These findings have important implications for understanding the molecular pathogenesis of neurodegenerative disorders and the therapeutic potential of mitochondria-targeted antioxidants.

## 2 Material and methods

### 2.1 Animals and treatment

Male wild-type (WT) B6CBAF1/J mice and male transgenic R6/2 mice (B6CBA-Tg(HDexon1)62Gpb/3J) expressing exon 1 of the human huntingtin gene with 120 ± 5 CAG were obtained from Charles River (Barcelona, Spain). R6/2 mice are an HD model with a rapid onset of symptoms, being the most frequently used in the pre-clinical settings [28,29]. Mice arrived at 4 weeks old and were housed in groups of 5 animals under controlled environment (12 light/dark cycle, 21±1 °C) with food and water *ad libitum*. Experiments were planned and performed according to the 3Rs principle (Replacement, Reduction and Refinement), which comprise the reduction of animal suffering and the number of mice used [30]. Sample size (*n* = 5, per group) was chosen according to calculation of the standardised effect size using rotarod data of WT and R6/2 mice described in the literature [31], for a significance level of 5% and a power of 90% (http://www.biomath.info/power/). All procedures were previously approved by the Institutional Animal Care and Use Committee from the Centre for Neuroscience and Cell Biology of the University of Coimbra and by the Portuguese Directorate-General for Food and Veterinary, and performed according to the European Union guidelines.

Treatment with 500 μM MitoQ complexed β-cyclodextrin (MitoQ Inc, Auckland) or vehicle (0.12% w/v β-cyclodextrin, Sigma-Aldrich) were administered *ad libitum* in drinking water, starting at 5 weeks of age. This treatment regime was previously shown safe and effective in mice [23,32]. Drug renewal, mouse weighing, and welfare monitoring were performed twice a week until the end of the experiments. Animals were euthanized by cervical dislocation at 11 weeks of age. Brain, liver and quadriceps muscle were immediately extracted, snap-frozen and stored at -80 °C.

### 2.2 Behavioural tests

Behavioural assays were performed between 5 and 11 weeks of age, in a sound-attenuated room under controlled temperature and low-intensity light. Mice were acclimated to the room in their home cages, for at least 2 h prior to testing, and were handled by the same person during the tests. The apparatuses were cleaned with 10% ethanol between animals.

#### 2.2.1 Grasping strength

Grasping strength was performed as previously described with minor adaptations [33]. Mice were allowed to grasp with their forepaws a metal grid, fixed to a 300 g weight on top of an electronic scales, while being held by the tail with increasing firmness, until they loosened the grid. The weight (g) change recorded in the scales was divided by the animal weight (g) and expressed as the grasping strength. This assay was repeated 10 times for each animal and the computed result was the average of the 5 trials with the highest values.

#### 2.2.2 Open field

Individual mice were gently positioned in the centre of an open field arena (38 × 38 cm) and allowed to move freely for 15 min, while being video-recorded. The ANY-MAZE software (Stoelting Co.) was used for mouse detection and tracking [34].

#### 2.2.3 Paw clasping

Clasping was assessed in mice suspended by the tail for 30 s [31]. Mice were scored as positive when at least one event of fore and/or hindlimb clasping was observed in the 30 s period.

#### 2.2.4 Pole test

The pole test was performed with a previously described modification for the R6/2 model [31]. Mice were placed head downward on the top of a vertical pole (50 cm in height and 1 cm in diameter) whose base was positioned in the home cage. The time required for mice to descend and reach the floor with all four paws was recorded. The task was repeated 3 times with 60 s rest in between. Each repetition had a maximum waiting period of 120 s. For each mouse, the lowest descending time out of the 3 repetitions was used for statistical analysis [33].

#### 2.2.5 Rotarod

Mice were placed on the cylinder at a constant rotation (4 rpm) (rotarod LE8200, Panlab). After 2 s at 4 rpm, the cylinder was progressively accelerated from 4 to 40 rpm over 5 min. Mice were assayed individually: if they fell before starting the acceleration, the assay could be repeated until a maximum of 3 trials. The assay ended when the mouse fell off the rod and the latency to fall was recorded [33].

### 2.3 Quantification of tissue MitoQ content

MitoQ content was quantified by liquid chromatography-tandem mass spectrometry (LC-MS-MS) in brain, liver and skeletal muscle. Tissue samples of ~ 50 mg in 250 μL of 95% acetonitrile and 0.1% formic acid were spiked with 50 pmol of d_15_-MitoQ and processed with a bead-based homogenizer (Precellys Evolution, Bertin Instruments - 6800 rpm, 3 × 20 s and pauses of 30 s). The homogenate was centrifuged at 16,000×*g* for 10 min and the supernatant collected. The pellet was re-extracted (resuspended in 95% acetonitrile and 0.1% formic acid and homogenised) and re-centrifuged once. Pooled supernatants were filtered (0.22 μm) and evaporated under a stream of nitrogen. Dried material was then resuspended in 100 μL of 60% acetonitrile/0.1% formic acid with vortexing and sonication. MitoQ standards were prepared in liver samples from untreated WT mice, spiked with 0–500 pmol MitoQ and 50 pmol of d_15_-MitoQ. MitoQ quantification was performed in a LC/MS/MS system comprising a Waters Quattro Ultima triple-quadrupole mass spectrometer, a binary pump (Model 1585; Jasco), an HTC-PAL autosampler (CTC-Analytics) and a Luna 5μm Phenyl–Hexyl column (1 × 50 mm, 5 μm) with a Phenyl–Hexyl guard column (2 × 4 mm; both from Phenomenex) as previously described [32].

### 2.4 Protein extraction

Brain (cortex), liver and muscle samples were homogenised (Precellys Evolution - 6800 rpm, 3 × 20s and pauses of 30s) in RIPA buffer (50 mM Tris, 150 mM NaCl, 1 mM EDTA, 1% NP-40 and 0.25% sodium deoxycholate; pH 7.4) containing protease inhibitors. Muscle samples required an additional 20 s of sonication to ensure homogenisation. Homogenates were incubated for 1 h in ice and centrifuged at 12,000×*g* for 10 min. When processing samples for Htt quantification, the centrifugation force was reduced to 300×*g* to avoid depositing Htt oligomers/aggregates in the pellet. Supernatants were collected and frozen. Protein was quantified by the Bradford protein assay (Bio-Rad).

### 2.5 Dot blot

For the Dot blot technique protein extracts were spotted in nitrocellulose membrane using a Bio-Dot Microfiltration Apparatus (Bio-Rad). To quantify carbonyl groups, sample derivatization was performed as we previously described [18]. Briefly, protein extracts containing 20 μg of protein were incubated with 200 μL of 12% sodium dodecyl sulfate (SDS) and 400 μL of 20 mM 2,4-dinitrophenylhydrazine hydrochloride (TCI Europe) for 30 min in the dark. After neutralization with 300 μL of 2 M Tris with 18% β-mercaptoethanol, samples containing 1 μg of protein were spotted on the membrane. To quantify ubiquitinated proteins, protein extracts containing 20 μg of protein were denatured at 95 °C for 5min in presence of 2% of SDS before spotting on the membrane. To quantify Htt, protein extracts containing 20 μg of protein were directly spotted on the membrane without derivatization or denaturation.

Spotted membranes were blocked with 5% bovine serum albumin (BSA) in PBST (phosphate buffer solution with 0.05% Tween 20) and incubated with primary antibodies (Htt: 1:1000 anti-N-terminal-Htt, CH00146, Coriell Institute; 1:1000 mEM48, MAB5374, Sigma-Aldrich; ubiquitin: 1:1000 anti-ubiquitin, sc-8017, Santa Cruz Biotechnology; carbonyl groups: 1:1000 anti-dinitrophenol, MAB2223, Sigma-Aldrich). After washing, membranes were incubated with suitable horseradish peroxidase-conjugated secondary antibodies (1:4000 anti-mouse, G-21040; or 1:4000 anti-rabbit, G-21234, Life Technologies) for 1 h, and imaged using a Chemiluminescent kit (Novex ECL, Life Technologies) and a ChemiDoc MP Imaging system (Bio-Rad). Membrane Coomassie staining was used for loading control. Densitometric analyses were performed with Image J (https://imagej.nih.gov/ij/).

### 2.6 Western blot

Western blot was performed as we previously described with some modifications [35]. Briefly, protein extracts were denatured at 95 °C for 5min in NuPAGE LDS sample buffer (Invitrogen) supplemented with NuPAGE sample reducing agent (Invitrogen). Protein extracts were loaded (25 μg) into 4–12% Bis-Tris gels (Invitrogen), electrophoresed at 200 V for 30 min and electroblotted to PVDF membranes using an iBlot-2 dry transfer device (Invitrogen). Membranes were blocked in PBST containing 5% of BSA and then incubated with primary and respective horseradish peroxidase-conjugated secondary antibodies. Detection and quantification were performed as described in section 2.5. Membrane Coomassie staining was used as loading control. Primary Antibodies: anti-Hsp60 (1:1000, sc-13115), anti-Hsp70 (1:1000, sc-66049), Hsp90 (1:1000, sc-13119), anti-SOD2 (1:500, sc-137254) and TFAM (1:750, sc-23588) from Santa Cruz Biotechnology; anti-SDHA (1:2000, ab14715) from Abcam; anti-SQSTM1/p62 (1:500, #5114) and anti-LC3A/B (1:1000, #4108) from Cell Signaling Technology; and anti-Hsp40 (1:1000, PA5-17382) from Invitrogen. Secondary Antibodies: horseradish peroxidase-conjugated anti-mouse (1:4000, G-21040) and anti-rabbit (1:4000, G-21234) from Invitrogen.

### 2.7 Immunoprecipitation and blue-native electrophoresis

Htt was detected in native conditions with the mEM48 antibody in brain and muscle extracts previously enriched by immunoprecipitation. Briefly, samples containing 200 μg of total protein were incubated (1 h) with SureBeads Protein G Magnetic beads (Bio-Rad) previously adsorbed (30 min) with anti-N-term-Htt (1:100, CH00146, Coriell Institute). Protein elution was performed in non-denaturing conditions using 20 mM glycine buffer (pH 2.0) [36]. After pH neutralization with 1 M phosphate buffer (pH 7.4), samples were supplemented with NativePAGE sample buffer (Invitrogen) and 10x Coomassie G-250 5% (w/v). Samples were loaded in a polyacrylamide gel (4% stacking, 4–10% resolving gradient) and run with the blue-native PAGE technique [37]: anode buffer (50 mM bis-tris, pH 7.0), cathode buffer (50 mM tricine, 15 mM bis-tris, pH 7.0). The run started with the cathode buffer containing 0.02% Coomassie blue G-250, which was replaced by a cathode buffer without Coomassie blue G-250 at the middle of the run. Proteins were transferred into PVDF membranes (overnight, 30 V, 4 °C; Mini Trans-blot Cell, Bio-Rad) in electroblotting buffer (50 mM tricine, 7.5 mM imidazole; pH 7.0). Membranes were then washed with methanol to reduce the Coomassie G-250 staining and blocked in PBST containing 5% of BSA. Htt was labelled using the mEM48 antibody (1:1000, MAB5374, Sigma-Aldrich). Detection and quantification were performed as described in section 2.5.

### 2.8 Statistical analysis

Data are from a minimum of 5 mice per condition and are plotted as mean ± standard error of the mean (SEM), unless otherwise stated in figure legends. The Shapiro-Wilk normality test was used to assess data distributions. Two-way ANOVA was used to test for interactions and main effects of the factors: genotype (WT, R6/2) *vs.* treatment (vehicle, MitoQ); and of the factors: tissue (brain, liver, muscle) *vs.* treatment. When two-factor interactions were significant, multiple comparisons among groups were performed with the Sidak's post-Hoc test. Correlations between normally distributed data were assessed with the Pearson correlation test. For all analyses, a *P* value under 0.05 was taken as statistically significant. Data analyses were performed with Prism 8.0 (GraphPad).

## 3 Results

### 3.1 R6/2 mice show impaired behaviour and MitoQ ameliorates loss of fine motor control

To investigate the role of aberrant ROS signalling in HD pathogenesis and, particularly, the therapeutic potential of treatment with a mitochondria-targeted antioxidant (MitoQ), we compared R6/2 HD mice with age-matched WT controls and tested for genotype × treatment interactions and main effects. The treatment regime with MitoQ (500 μM *ad libitum*) *vs.* vehicle control was selected based on previous *in vivo* data on MitoQ safety and efficacy [23,32]. We first characterized the HD phenotype of R6/2 mice by comparing their weight gain over time with that of age-matched WT controls, and by performing behavioural tests [31,38].

**Fig. 1:**
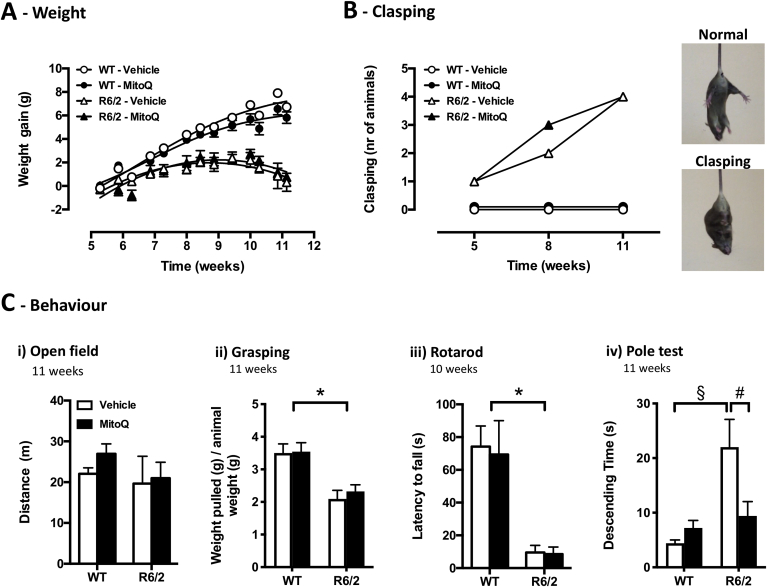
Weight and behavioural phenotypes of R6/2 mice and the effect of MitoQ. **(A)** Weight gain over time in wild-type (WT; circles) and R6/2 (triangles) mice under vehicle (white) or MitoQ (500 μM; black) treatment conditions. **(B)***Left*, paw clasping scores at 5, 8 and 11 weeks; *Right*, representative images of normal paw positioning vs. paw clasping under tail-suspension. **(C)***i*) Travelled distance in the open-field test; *ii*) weight pulled per mouse weight in the grasping strength test; *iii*) latency to fall from the accelerating rotarod; *iv*) descending time in the pole test. *n* = 5; *P < 0.05, genotype effect; ^§^*P* < 0.05 *vs*. WT-vehicle; ^#^*P* < 0.05 *vs*. R6/2-vehicle.

R6/2 mice exhibited a progressive HD-like phenotype, characterized by reduced weight gain and motor dysfunction, as previously described [39,40]. While WT mice gained weight steadily over time, R6/2 mice gained weight only until 8 weeks of age (Fig. 1A) and showed a progressive increase of paw clasping scores (Fig. 1B). This motor dysfunction of R6/2 mice was not so severe as to impair spontaneous locomotion in the open field test, where R6/2 mice travelled similar distances to WT mice even at 11 weeks (Fig. 1Ci). However, the R6/2 mice behavioural impairments manifested with lower grasping strength per animal weight, shorter latency to fall from a rotating rod, and increased descending time in a vertical pole (Fig. 1*Cii-iv*).

When compared with vehicle treatment (control), treatment with MitoQ evoked no changes in weight gain or clasping phenotypes (Fig. 1A,B). Also, MitoQ did not alter the grasping strength or the latency to fall (Fig. 1Ci-iii). However, MitoQ significantly reduced the time required by R6/2 mice to descend the vertical pole (Fig. 1Civ). These results indicate that treatment with MitoQ is only partially effective in rescuing HD phenotypes, but may hold potential to ameliorate loss of fine motor control in HD, as suggested by improved performance in the vertical pole test.

### 3.2 MitoQ reduces oxidative stress biomarkers, particularly in the R6/2 muscle

**Fig. 2:**
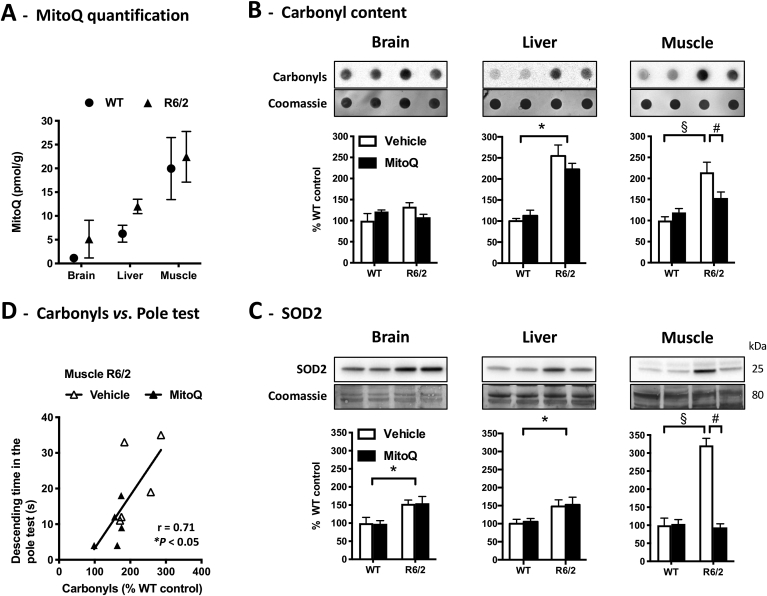
MitoQ tissue distribution and effects on oxidative stress biomarkers. **(A)** Quantification of MitoQ levels in brain, liver and muscle of MitoQ-treated WT and R6/2 mice by LC-MS-MS; *n* = 3. **(B, C)** Oxidative stress biomarkers in brain, liver and muscle of WT and R6/2 mice treated with vehicle or MitoQ: protein carbonyl groups measured by dot blot; SOD2 levels measured by western-blot. Data are in percentage of the mean of WT-vehicle (control); *n* = 5; *P < 0.05, genotype effect; ^§^*P* < 0.05 *vs*. WT-vehicle; ^#^*P* < 0.05 *vs*. R6/2-vehicle. **(D)** Correlation between the descending time in the pole test and muscle carbonyl content for R6/2 mice.

To characterize the molecular pathogenesis in the HD mice, to assess MitoQ distribution, and to test for MitoQ effects upon oxidative stress markers, we analysed samples from R6/2 and WT tissues. Brain, muscle, and liver were chosen given that HD is a neurodegenerative disorder with muscular atrophy and metabolic dysfunction [10,41]. The muscle of R6/2 and WT mice presented the highest levels of MitoQ, about twice the levels found in the liver, whereas the brain presented the lowest amounts, indicating a low brain bioavailability of MitoQ (Fig. 2A).

We measured protein carbonylation as a marker of oxidative damage [42] and levels of mitochondrial superoxide dismutase (SOD2), a marker of the antioxidant response to oxidative stress [43]. R6/2 mice presented higher levels of protein carbonyls and of SOD2 than WT mice: carbonyls were significantly elevated in liver and muscle (Fig. 2B), whereas SOD2 levels were significantly elevated in all tissues analysed (Fig. 2C). Focusing on R6/2 mice, treatment with MitoQ induced an overall decrease in protein carbonyls (tissue × treatment interaction: *F*_(2,24)_ = 0.615, *P* = 0.549; treatment effect: *F*_(1,24)_ = 7.74, *P* = 0.010). However, the effect of MitoQ was particularly evident in muscle, where it reduced not only protein carbonyls but also SOD2 levels (Fig. 2B,C). These results suggest that MitoQ, a mitochondria-targeted chain-breaking antioxidant [21], reduces oxidative stress in R6/2 mice, particularly in muscle where the compound has higher bioavailability. This raises the mechanistic hypothesis that MitoQ improves fine motor performance in the pole test by reducing oxidative damage in the R6/2 muscle. To test this hypothesis, we plotted levels of protein carbonyls in R6/2 mice against the respective scores in the pole test (Fig. 2D). The correlation between both values strongly suggests that the levels of carbonyls in muscle predict pole test performance. Moreover, these data support the mechanistic hypothesis that high muscle bioavailability of MitoQ reduces oxidative damage to muscle and thereby ameliorates fine motor control in R6/2 mice.

### 3.3 R6/2 mice have increased levels of mitochondrial and cytosolic chaperones

To examine the molecular changes in proteostasis effectors in the HD mice, and also attending to the mitochondrial targeting of MitoQ [21], we measured key mitochondrial biomarkers and molecular chaperones. Concerning mitochondria, we focused on SDHA (succinate dehydrogenase complex, subunit A), TFAM (mitochondrial transcription factor A), and Hsp60 (mitochondrial chaperone) as indicators of mitochondrial mass, biogenesis, and proteostasis, respectively [[44], [45], [46]]. Concerning cytosolic chaperones, we focused on Hsp90, Hsp70 and its co-chaperone Hsp40 as key effectors of the proteostasis network involved in protein stabilization, refolding, or targeting for degradation [47].

**Fig. 3:**
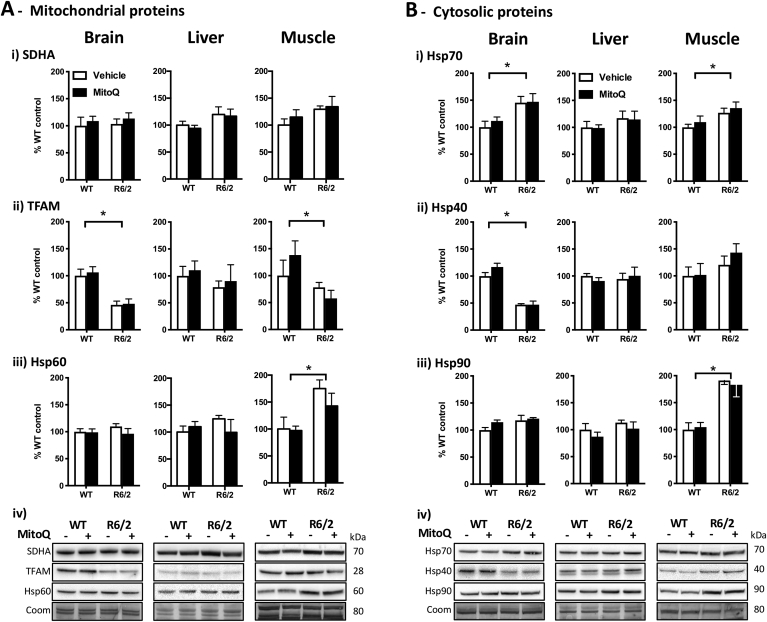
Levels of mitochondrial biomarkers and chaperones in WT and R6/2 mice. Western blot quantifications in brain, liver and muscle samples from WT and R6/2 mice treated with vehicle or MitoQ. **(A)** Mitochondrial biomarkers: *i*) SDHA, succinate dehydrogenase complex, subunit A; *ii*) TFAM, mitochondrial transcription factor A, *iii*) Hsp60; and *iv*) representative blots. **(B)** Cytosolic chaperones: *i*) Hsp70; *ii*) Hsp40; *iii*) Hsp90; and *iv*) representative blots. Coom – Coomassie staining loading control. Data are in percentage of the mean of WT-vehicle (control); *n* = 5; *P < 0.05, genotype effect.

The levels of SDHA were similar in R6/2 and WT mice, independently of the tissue (Fig. 3Ai), which suggests no major differences in mitochondrial mass between genotypes. In contrast with similar SDHA levels, however, TFAM and Hsp60 levels were significantly altered (Fig. 3A). The brain and muscle of R6/2 mice displayed lower levels of TFAM (Fig. 3Aii), suggesting a reduction of the mitochondrial DNA transcription and, consequently, of the mitochondrial biogenesis [48]. Regarding mitochondrial proteostasis, the muscle of R6/2 mice presented higher levels of the Hsp60 chaperone (Fig. 3Aiii), indicating an activation of the mitochondrial unfolded protein response (UPR^mt^), which is a transcriptional response of the cell to a mitochondrial stress [49].

Concerning the cytosolic proteostasis network, the brain and muscle from R6/2 mice presented significant differences from WT mice in the levels of molecular chaperones (Fig. 3B). This shows that the R6/2 brain and muscle are under proteotoxic stress, unlike the liver where the lack of differences in chaperone levels suggests no activation of an unfolded protein response. Specifically, the R6/2 brain and muscle presented higher levels of Hsp70 and, interestingly, the co-chaperone Hsp40 was significantly decreased in the R6/2 brain, whereas the Hsp90 chaperone was significantly elevated in the R6/2 muscle (Fig. 3B). These results suggest that there is altered mitochondrial and cytosolic proteostasis in R6/2 mice, with an activation of the unfolded protein responses. While treatment with MitoQ evoked no significant changes in the levels of chaperones, we cannot exclude the hypothesis that reducing mitochondrial oxidative damage with MitoQ may attenuate the proteotoxic stress in the mitochondria (MitoQ treatment effect upon Hsp60 levels across R6/2 tissues: *F*_(1,24)_ = 3.22, *P* = 0.086).

### 3.4 R6/2 mice show increased protein ubiquitination and autophagy induction in muscle, which is decreased by MitoQ

Following evidence for oxidative stress and altered chaperone levels in R6/2 mice, we further investigated how changes in redox signalling might influence the proteostasis network, now focusing on protein degradation pathways. Pursuing the hypothesis that the UPS and autophagy pathways are under redox regulation [8,50], we examined the levels of ubiquitinated proteins and autophagy markers in R6/2 and WT mice, and how these were affected by treatment with the mitochondria-targeted antioxidant MitoQ.

**Fig. 4:**
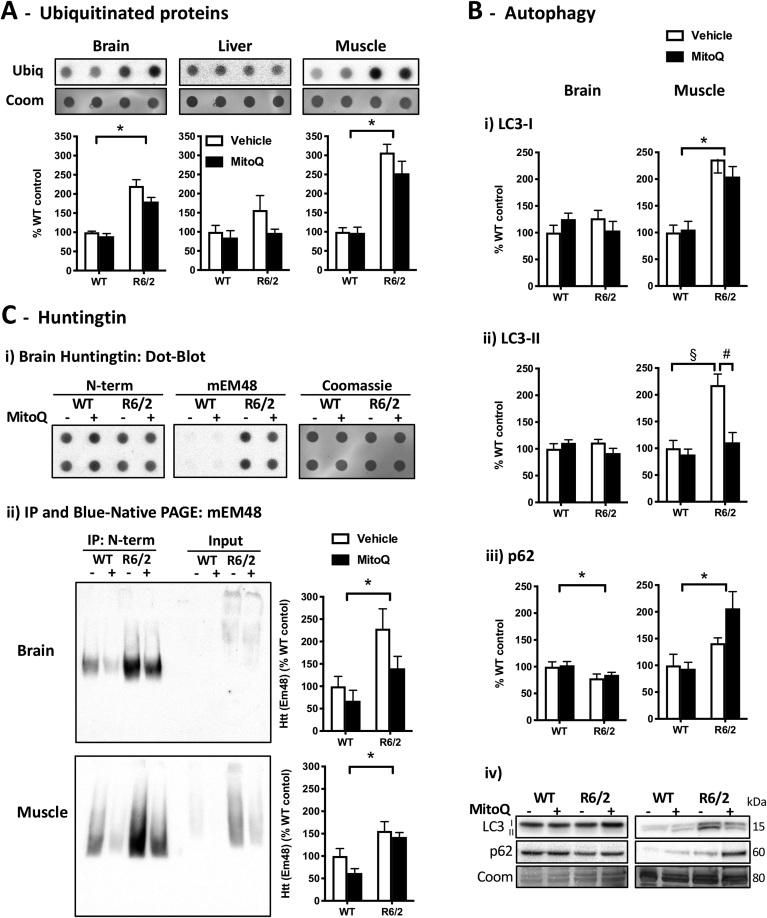
Levels of ubiquitinated proteins, autophagy biomarkers and huntingtin. Proteostasis markers in tissue samples from WT and R6/2 mice treated with vehicle or MitoQ. **(A)** Dot blot quantification of ubiquitinated proteins (ubiq); *Coom*, Coomassie staining loading control. (**B**) Western blot quantification of autophagy biomarkers: *i*) LC3-I; *ii*) LC3-II; *iii*) p62; and i*v*) representative blots. **(C)** Quantification of huntingtin (Htt): *i*) dot blot of native (non-denatured) brain extracts using an N-terminal (N-term) Htt antibody or the anti-Htt mEM48 antibody; *ii*) immunoprecipitation (IP) with anti-N-terminal Htt in brain and muscle samples, followed by blue-native page using the mEM48 antibody. Data are in percentage of the mean of WT-vehicle (control). *n* = 5; *P < 0.05, genotype effect; ^&^*P* < 0.05, treatment effect; ^§^*P* < 0.05 *vs*. WT-vehicle, ^#^*P* < 0.05 *vs*. R6/2-vehicle.

The levels of ubiquitinated proteins were significantly elevated in the brain and muscle, but not in the liver of R6/2 mice when compared with WT mice (Fig. 4A). This tissue-specific profile agrees with the observed changes in molecular chaperones (Fig. 3B) and indicates that the proteotoxic stress is higher in the R6/2 brain and muscle than in the liver. We next studied autophagy markers, particularly LC3-I, LC3-II and p62. In the process of autophagosome formation, LC3-I converts into the lipidated form LC3-II, which is a marker of completed autophagosomes [51]. The autophagic adaptor p62 functions as a link between LC3 and ubiquitinated substrates, and is itself a substrate for autophagic degradation [51,52].

The levels of LC3-I and LC3-II in the R6/2 brain were not significantly different from WT, albeit the decrease in p62 levels in the R6/2 brain suggests there could be a modest increase in its autophagic degradation (Fig. 4B). In the brain, the levels of autophagic markers were unaffected by treatment with MitoQ, in agreement with its reduced brain bioavailability (Fig. 2A). In contrast, the levels of LC3-I and LC3-II were significantly elevated in the R6/2 muscle in comparison with WT (Fig. 4B), suggesting more autophagosomes. In the R6/2 muscle, treatment with MitoQ decreased LC3-II levels without decreasing LC3-I and p62 levels (Fig. 4B, R6/2 muscle: MitoQ *vs.* vehicle), whereas in WT muscle MitoQ did not change autophagy markers (Fig. 4B, WT muscle: MitoQ *vs.* vehicle). Taken together with increased carbonyl levels in the R6/2 muscle (Fig. 2B), these results suggest that there is a ROS-mediated autophagy induction in the R6/2 muscle, which is attenuated by the antioxidant MitoQ resulting in decreased LC3-I to LC3-II conversion.

**Fig. 5:**
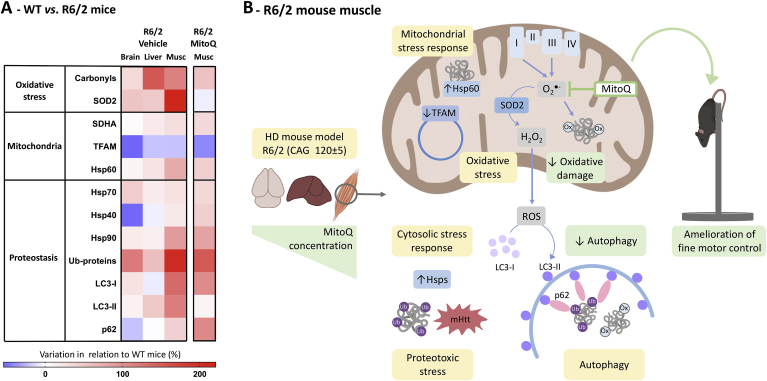
Interplay between ROS signalling and proteostasis: Effects of MitoQ. (**A**) Heat map summarising the biomarker profile in tissues from R6/2 HD mice in relation to WT under vehicle-treatment conditions, and the effects of MitoQ treatment in R6/2 muscle. (**B**) Mechanistic pathways for MitoQ effects in the R6/2 mouse muscle. The mitochondria-targeted antioxidant MitoQ accumulates in mitochondria where it interacts with ROS, such as superoxide [21], reducing overly active redox signalling pathways and thereby attenuating oxidative damage and the redox-regulated induction of autophagy [9].

Given the observed changes in protein degradation pathways in the R6/2 brain and muscle, we next examined the levels of Htt in these tissues and whether these were modulated by MitoQ. To detect Htt, we first performed dot-blots using brain samples in native (non-denatured) conditions. In these native dot-blots the anti-N-terminal Htt antibody labelled both WT and R6/2 samples, whereas the mEM48 antibody preferentially labelled the R6/2 samples (Fig. 4Ci). Indeed, although mEM48 was generated by using the first 256 amino acids of Htt without the polyQ tract, mEM48 preferentially recognizes Htt aggregates [[53], [54], [55]]. To quantify Htt, we performed an immunoprecipitation with the anti-N-terminal antibody and ran the enriched samples in blue-native PAGE (with Coomassie blue G-250 to confer a net negative charge to proteins while preserving their native state [37]). In the blue-native PAGE, mEM48 yielded a significantly stronger labelling in the immunoprecipitated samples of R6/2 *vs.* WT mice (Fig. 4Cii). Treatment with MitoQ induced no significant changes in the overall labelling achieved with mEM48. These results suggest that treatment with MitoQ does not significantly alter the levels of Htt. As summarized in Fig. 5, the amelioration of fine motor performance in R6/2 mice treated with MitoQ is likely due to a combination of reduced oxidative damage and attenuation of ROS-induced muscle autophagy.

## 4 Discussion

The bidirectional interplay between redox signalling and proteostasis is an active field of research. Disturbances in these processes are associated with neurodegenerative disorders and may hold potential for therapeutic targeting. Here we investigate this interplay and perform the first *in vivo* testing of the mitochondria-targeted antioxidant MitoQ in HD mice. We found widespread oxidative stress and alterations in the proteostasis network that differ across HD mice tissues. Data highlight the relevance of peripheral HD pathology, particularly in muscle where MitoQ may have beneficial effects.

### 4.1 R6/2 mice model systemic HD with widespread oxidative stress and tissue-specific alterations in the proteostasis network

HD is primarily known as a neurodegenerative disorder. However, patients often present weight loss, metabolic dysfunction, skeletal muscle atrophy, and osteoporosis [41,56], highlighting the systemic nature of HD [10,11]. Recent studies with neurons, muscle cells, and hepatocytes derived from HD stem cells support the tissue-specificity of molecular HD phenotypes [57]. Thus, to address HD as a systemic disease, we studied the brain, skeletal muscle, and liver from R6/2 HD mice.

Impaired redox signalling is a hallmark of HD, with patients and animal models exhibiting oxidative damage to proteins, lipids, and DNA [7,[15], [16], [17],^58^]. In the present study, R6/2 mice with 11 weeks presented increased levels of the mitochondrial antioxidant enzyme SOD2 in the brain, liver, and muscle, indicating a widespread response to oxidative stress. Protein carbonyls were also elevated in R6/2 liver and muscle, suggesting that muscle dysfunction contributes to the motor behaviour phenotypes. In the R6/2 brain, however, carbonyls were not yet significantly elevated, possibly because the brain susceptibility to oxidative damage [59] was still compensated by its effective antioxidant defences and proteostatic machinery that handle oxidized proteins [60].

The proteostasis network includes molecular chaperones and protein clearance pathways, such as the UPS and autophagy. Under physiological conditions, the proportion of central chaperones such as Hsp70, Hsp40 and Hsp90 is thought to remain relatively constant in all tissues. Recent data, however, show that the proteostasis network can be tailored to specific proteomes, being controlled by neuronal activity and inter-tissue communication [61]. Here, we measured protein levels of central chaperones and found them significantly altered in the brain and muscle of R6/2 mice, together with increased ubiquitinated proteins. These alterations in proteostasis were tissue-specific, being absent in the liver. Accordingly, elevations in chaperone mRNAs were previously found in skeletal muscle, but not in liver from R6/2 mice [62]. We also found pronounced changes in autophagy markers in the R6/2 muscle, which together with increased SOD2 and carbonyls support the hypothesis that an interplay between abnormal redox signalling and proteostasis in muscle contributes to motor impairment. These data support skeletal muscle pathology as a hallmark of HD [29] and a therapeutic target for redox and proteostasis modulators.

### 4.2 R6/2 mice display mitochondrial and cytosolic unfolded protein responses

Mitochondrial stress conditions, such as those triggered by excessive ROS or the accumulation of misfolded proteins, can elicit a transcriptional response called the UPR^mt^ [63]. The UPR^mt^ promotes mitochondrial recovery by decreasing general translation while inducing mitochondrial proteases and chaperones, such as Hsp60 [49,64]. It remains uncertain whether the UPR^mt^ is activated in HD. Contrasting data from HD cell models either support UPR^mt^ activation – by showing increases in mitochondrial chaperone levels [65]; or suggest that mHtt inhibits the UPR^mt^ – by showing impaired mRNA stability of the ABCB10 transporter whose deletion decreases mitochondrial chaperone levels [66]. Given the tissue-specificity of proteostatic responses [61], studies in animal models may help clarify the UPR^mt^ status in HD.

Our data in R6/2 mice suggest an activation of the UPR^mt^ specifically in muscle, characterized by increased levels of Hsp60 and decreased levels of TFAM. Increased Hsp60 contributes to the UPR^mt^ by promoting protein folding in mitochondria, whereas decreased TFAM may contribute to the UPR^mt^ by limiting mitochondrial protein synthesis [45]. Two complementary mechanisms may explain decreased TFAM levels in the UPR^mt^: (1) increased TFAM degradation by the mitochondrial LON protease [67,68]; and (2) repression of NRF1-mediated TFAM transcription [69]. We also observed decreased TFAM in R6/2 brain samples, which is compatible with the transcriptional attenuation detected in brain samples of HD patients [70], and could be part of a pro-survival unfolded protein response. In fact, the experimental activation of the UPR^mt^ was found protective in disease models [71,72], including HD cell models where UPR^mt^ activation by GRP75 knockdown reduced mHtt aggregation [65].

Recent studies suggest that the UPR^mt^ can coordinate with UPRs in other compartments, namely via changes in lipid biosynthesis that signal to the nucleus and induce the expression of cytosolic chaperones [64,65]. Here, we found increased levels of Hsp70 in the brain and muscle of R6/2 mice, together with increased ubiquitinated proteins, suggesting the activation of a cytosolic UPR in response to mHtt proteotoxicity. Despite the increase in Hsp70, its activity may be compromised in the R6/2 brain where we also found decreased levels of Hsp40. Indeed, Hsp40 is a limiting factor for a trimeric chaperone complex, including Hsp70 and Hsp110, which disaggregates mHtt fibrils [73]. Previous studies in R6/2 mice have found age-dependent decreases in the levels of brain chaperones [74,75], their sequestration into mHtt aggregates [76,77], and a phenotypic rescue with Hsp40 gene therapy [[78], [79], [80]].

In the R6/2 muscle, in addition to increased Hsp70 we found increased Hsp90, supporting the hypothesis that mHtt can induce the proteostasis network in peripheral tissues such as skeletal muscle [62]. Interestingly, it was recently proposed that Hsp90 and Hsp70 interact with the ATP-gated ion channel P2RX7 to mediate autophagy induction in myoblasts [81]. This raises the hypothesis that increased cytosolic chaperones in the R6/2 muscle might play a role in autophagy induction in this tissue, which has been associated with muscle wasting [82].

### 4.3 Treatment with MitoQ ameliorates fine motor control and attenuates ROS-induced muscle autophagy in R6/2 mice

Oxidative stress is associated with neurodegenerative diseases [2], including HD [15], however, classical antioxidants have failed to benefit HD patients [[83], [84], [85]]. This could be due to lack of selectivity or disruption of redox signalling [85], being possible that antioxidants that selectively target mitochondrial ROS might provide clinical benefit [3,4]. Accordingly, the mitochondria-targeted antioxidant MitoQ reduced oxidative damage and ameliorated disease phenotypes in animal models of Parkinson's [25], Alzheimer's [26,86], ALS [24], and spinocerebellar ataxia [23]. Concerning HD models, MitoQ was tested *in vitro* in ST*Hdh*^Q111^ cells where it reduced markers of oxidative damage and the mutant-huntingtin-induced mitochondrial toxicity and synaptic damage [87]. While these studies suggest protective effects for MitoQ, its impact on proteostasis was not assessed. Here, we investigated the interplay between redox signalling and proteostasis in R6/2 mice, using MitoQ as a redox-modulator and assessing its *in vivo* therapeutic potential in HD.

Treatment with MitoQ was only partially effective against the behavioural phenotypes of R6/2 mice, but significantly improved their fine motor control in the pole test. The pole test is a complex task that has been previously used to monitor motor and sensorimotor impairments in rodents [38]. Since pole test performance correlated with the levels of muscle carbonyls and these were significantly decreased by MitoQ, together with SOD2 levels, these data suggest that MitoQ ameliorates fine motor control by reducing oxidative damage in muscle. Indeed, the high bioavailability of MitoQ in muscle contrasts with its low brain bioavailability, suggesting peripheral rather than central nervous system effects. These findings support the hypothesis that redox interventions can improve muscle performance [88] and highlight the importance of peripheral therapeutics in HD.

The interplay between redox signalling and proteostasis is a topic under active discussion. ROS can modulate proteostasis effectors and, in turn, imbalances of proteostasis regulate redox homeostasis. Interestingly, this bidirectional regulation may occur across neighbouring cells and tissues, with proteostasis imbalances in neurons affecting the redox state of muscle cells and vice versa [5]. Although ROS can induce and regulate the activity of molecular chaperones [6,7] and the UPS [8,49], we found no significant treatment effect with MitoQ upon the levels of molecular chaperones, which remained altered in R6/2 tissues together with elevated levels of ubiquitinated proteins. However, the treatment with MitoQ significantly attenuated ROS-induced autophagy in the R6/2 muscle, where MitoQ has higher bioavailability.

ROS are early inducers of autophagy [9,50], and untargeted antioxidants are known to inhibit autophagy [27]. Recent *in vitro* studies in C2C12 myoblasts treated with the mitochondria-targeted MitoQ (≤0.5 μM) found that MitoQ attenuated autophagy by suppressing mitochondrial ROS production, which reduced mTOR and ULK1 phosphorylation, expression of Atg proteins, and the conversion of LC3-I to LC3-II [89]. In the present *in vivo* study, using a previously reported safe and effective dose [23,32], we found that MitoQ reduced the elevated LC3-II levels in the R6/2 muscle, without decreasing its p62 levels, and also without changing autophagy markers in WT muscle. This suggests that MitoQ reduces ROS-mediated autophagy induction in the R6/2 muscle. Importantly, this effect of MitoQ was selective for muscle, where MitoQ has higher bioavailability, and MitoQ did not change autophagic markers in the brain, where it has lower bioavailability. Thus, although autophagy is an important pathway to clear misfolded proteins, its excessive ROS-mediated induction in muscle appears maladaptive, so that selective autophagy attenuation with MitoQ might be beneficial to tackle peripheral HD symptoms.

## 5 Conclusion

Data from this study support the hypothesis that abnormal redox signalling in muscle contributes to altered proteostasis and motor impairment in HD. Moreover, this first *in vivo* study with the mitochondria-targeted redox modulator MitoQ in a HD mouse model shows that it can rescue fine motor control through its antioxidant effects at peripheral level. The high bioavailability of MitoQ in muscle and the attenuation of ROS-induced autophagy in this tissue could be advantageous to ameliorate muscle wasting, whereas low brain bioavailability of MitoQ limits the risk of impairing neuronal protein clearance mechanisms. This study also suggests that chaperone-mediated cytosolic and mitochondrial unfolded protein responses are active in the R6/2 HD mice and unimpaired by MitoQ treatment. MitoQ thus holds potential as an adjuvant treatment for peripheral HD symptoms. Future studies are now warranted with mitochondria-targeted antioxidants in slow progressive HD models to examine the potential long-term amelioration of disease phenotypes. Moreover, these data suggest that mitochondria-targeted antioxidants warrant further investigation in the context of neuromuscular disorders.

## Author contributions

BRP performed the majority of experiments, data analysis, and literature search. Behavioural experiments: BRP, AID, PMC. Molecular Biology: BRP. Manuscript writing: BRP and JMAO. Experimental design, data analysis, and discussion: BRP, AID, PMC, PIM, MPM, JMAO.

## Declaration of competing interest

MPM holds patents in the development of MitoQ as a therapy.
